# *Closo*- or *Nido*-Carborane Diphosphane as Responsible for Strong Thermochromism or Time Activated Delayed Fluorescence (TADF) in [Cu(N^N)(P^P)]^0/+^

**DOI:** 10.1021/acs.inorgchem.1c03092

**Published:** 2021-11-23

**Authors:** Adrián Alconchel, Olga Crespo, Pilar García-Orduña, M. Concepción Gimeno

**Affiliations:** Departamento de Química Inorgánica, Instituto de Síntesis Química y Catálisis Homogénea (ISQCH). Universidad de Zaragoza-CSIC, E-50009 Zaragoza, Spain

## Abstract

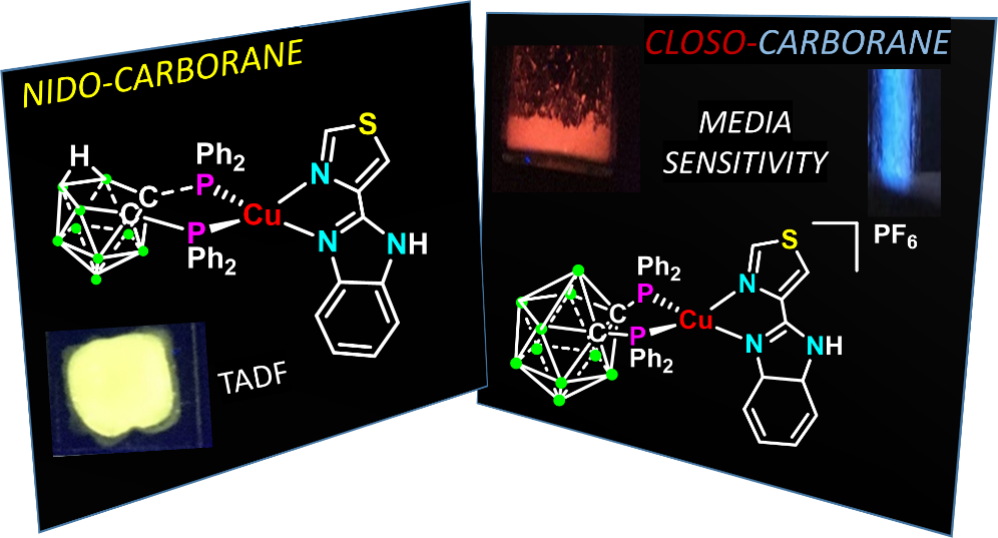

*Ortho*-*closo* or *ortho-nido*-carborane-diphosphanes have been selected to prepare the heteroleptic cationic or neutral [Cu(N^N){(PPh_2_)_2_C_2_B_10_H_10_}]PF_6_ (**1**) and [Cu(N^N){(PPh_2_)_2_C_2_B_9_H_10_}] (**2**) [N^N = 2-(4-thiazolyl)benzimidazole], respectively. Complexes **1** and **2** display very different emissive behavior. Neutral complex **2** exhibits TADF (time activated delayed fluorescence) which has been studied both as powder and PMMA composite with similar Δ*E*(S_1_ – T_1_), τ(T_1_), and τ(S_1_) in both phases. Cationic complex **1** displays a much lower quantum yield than **2** and does not show TADF, but it exhibits a significant thermochromic luminescence, and its emission is very dependent on the medium. Theoretical studies show that metal–ligand (M–diphosphane) to ligand (L′, diimine) transitions, MLL′CT, are responsible of the transitions which originate the emissive properties, but with very different contribution of the copper center, carborane cluster, and diphosphane phenyl rings for **1** and **2**.

## Abstract 2

*Ortho*-*closo* or *ortho*-*nido*-carborane-diphosphanes have been selected to prepare [Cu(N^N){(PPh_2_)_2_C_2_B_10_H_10_}]PF_6_ (**1**) and [Cu(N^N){(PPh_2_)_2_C_2_B_9_H_10_}] (**2**) [N^N = 2-(4-thiazolyl)benzimidazole]. Complexes **1** and **2** display different emissive behaviors. Neutral complex **2** exhibits TADF which has been studied both as a powder and PMMA composite with similar Δ*E*(S_1_ − T_1_), τ(T_1_), and τ(S_1_) in both phases. Complex **1** displays much lower quantum yield than **2** and does not show TADF, but it exhibits a thermochromic luminescence with emission dependent on the medium.

## Introduction

The synthesis and study of heteroleptic copper complexes [Cu(N^N)(P^P)]^*n*^ (*n* = 0, 1) represents a growing field in the study of emissive coordination complexes.^1−12^ One evident reason is the use of a cheaper metal (Cu) compared to those widely selected for the design of phosphors for emissive devices, such as Ir, Pt, or Au. Another relevant reason is related to their composition, which combines a diimine and a diphosphane. Substitution of a mostly planar diimine by a nonplanar ligand, a diphosphane, in the heteroleptic species diminishes the flattening distortion of the tetrahedral geometry in the excited state, compared with the homoleptic bis(diimine) copper complexes. This flattening distortion enables quenching mechanisms of the luminescence. ISC (intersystem crossing) should be increased by avoiding flattening distortion. As a consequence, in these systems, phosphorescence and TADF (time activated delayed fluorescence)^13^ should be favored. Both emissive mechanisms lead to an increment in the number of useful transitions for an emissive device.

These facts have prompted an important effort in the synthesis and study of heteroleptic copper complexes [Cu(N^N)(P^P)]^0/+^ for which the emission energies mostly lie in the yellow-green region. Among them, neutral complexes are still scarcely represented,^14−18^ and some of them exhibit TADF.^19^ Studies of composites are growing,^20−25^ also including those in which these derivatives are tested in OLED devices,^26,27^ revealing the potential of these systems.

Carborane diphosphanes are rigid electron-withdrawing ligands. Their rigidity could avoid flattening distortion, and the electron-withdrawing characteristics could affect the copper diamine bond, as the electron density at copper would be diminished, due to the electron-withdrawing effect of the carborane ligand, compared with other diphosphanes. Copper [Cu(N^N)(dppnc)][dppnc^–^ = (7,8-(PPh_2_)_2_-1,2-C_2_B_9_H_10_)^−^] complexes have been reported with different substituted phenanthroline diimine ligands.^14,28,29^ These complexes exhibit TADF, and some of them have been tested as dopants in the design of OLEDs. The influence of the partial degradation of the carborane cage in these properties has not been analyzed.

We have previously reported that the *nido* nature of the carborane diphosphane plays a relevant role in the luminescent properties in three coordinated group 11 complexes.^30,31^ Thus, we wonder if partial degradation of the carborane cage could also play such a key role in the TADF behavior of heteroleptic four coordinated copper derivatives. In addition, as most of the reported works display analysis of TADF for powder samples, our aim is also to compare τ(S_1_), τ(T_1_) (lifetime values of the first excited singlet and triplet, respectively) and Δ*E*(S_1_ – T_1_) (energy gap between the first excited singlet and triplet) in solid and PMMA films at 5% wt.

Here we report on the synthesis of two copper complexes with the diimine ligand 2-(4-thiazolyl)benzimidazole (N^N) and *ortho-*carborane diphosphanes: the cationic compound [Cu(N^N)(dppcc)]PF_6_**1** with the *closo*-diphosphane (PPh_2_)_2_-1,2-C_2_B_10_H_10_ (dppcc) and the neutral complex [Cu(N^N)(dppnc)] (**2**) with the *nido*-diphosphane [7,8-(PPh_2_)_2_-7,8-C_2_B_9_H_10_]^−^(dppnc), which display very different emissive behaviors. The emissive TADF behavior of **2** has been studied both as solid powder and as PMMA composite material.

## Discussion

### Synthesis and Characterization

**Scheme 1:**
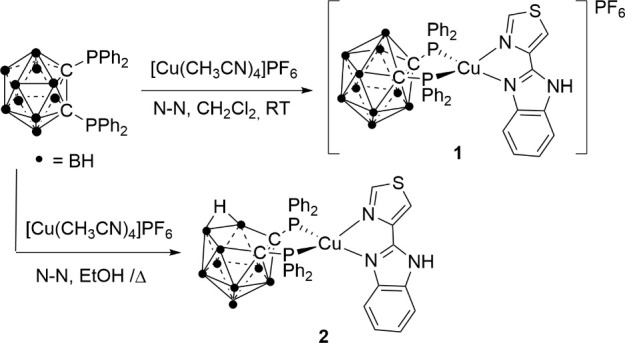
Synthetic Routes for Complexes **1** and **2**

Reaction of [Cu(CH_3_CN)_4_]PF_6_ with the carborane diphosphane 1,2-(PPh_2_)_2_-1,2-C_2_B_10_H_10_ and further addition of 2-(4-thiazolyl)benzimidazole (N^N) affords a cationic (**1**) or neutral (**2**) copper(I) complex (Scheme 1) depending on the solvent (dichloromethane or ethanol), respectively.

Complexes **1** and **2** have been characterized by NMR spectroscopy, showing a unique resonance for the ^31^P{^1^H} NMR spectra. A broad signal at −2 ppm in the ^1^H NMR spectrum, corresponding to the bridging H hydrogen, proves the partial degradation of the carborane cage in **2**.

**Figure 1:**
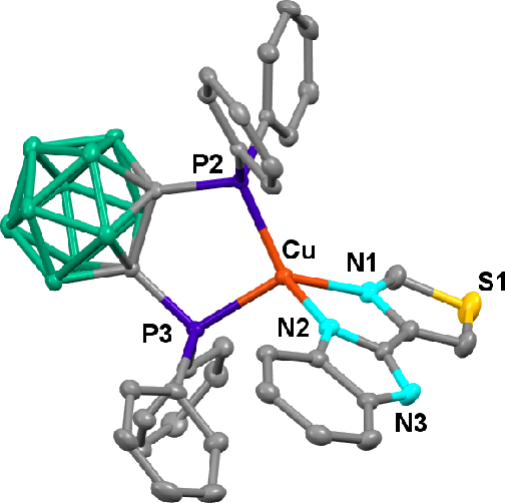
ORTEP diagram of the cation of compound **1**. Ellipsoids represent 50% probability. Hydrogen atoms have been omitted for the sake of clarity. Bond distances (Å) and angles (deg): Cu–P2 2.2242(11), Cu–P3 2.2398(11), Cu–N1 2.073(3), Cu–N2 2.057(3), N2–Cu–N1 81.60(13), P2–Cu–P3 98.18(4).

The crystal structure of compound **1** has been elucidated by X-ray crystal diffraction (Figure 1). The copper center displays a distorted tetrahedral environment due to the small bite angles of both chelating ligands, N2–Cu–N1 81.60(13)°, P2–Cu–P3 98.18(4)°, with the one with the N^N ligand being narrower. The angle between N1–Cu–N2 and P2–Cu–P3 planes is 82.70°.

**Figure 2:**
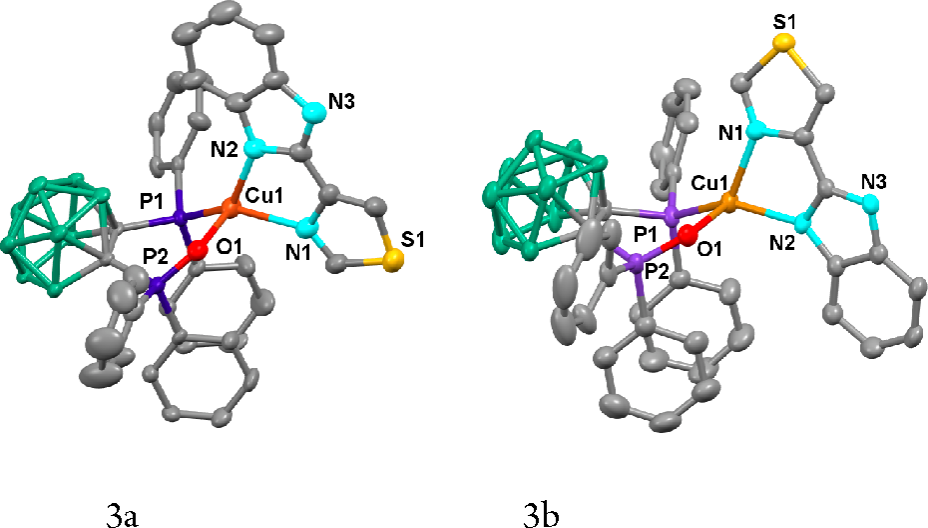
ORTEP diagram of the isomers of compound **3**. Ellipsoids represent 50% probability. Hydrogen atoms have been omitted for the sake of clarity. Bond distances (Å) and angles (deg): **3a** (acetone solvate) [Cu1–O1 2.165(4), Cu1–P1 2.1665(13), Cu1–N1 2.162(4), Cu1–N2 2.020(4), N2–Cu1–N1 80.20(16)]; **3b** (dichloromethane solvate) [Cu1–O1 2.234(4), Cu1–P1 2.1759(14), Cu1–N1 2.050(4), Cu1–N2 2.148(4), N2–Cu1–N1 80.26(17)].

Unexpectedly, the X-ray diffraction analysis of one crystal obtained from slow diffusion of *n*-hexane over an acetone solution of **2** leads to the structure shown in Figure 2, which corresponds to [Cu(N^N)(Odppnc)] (**3a**) [Odppnc = (1-(OPPh_2_)-2-(PPh_2_)-1,2-C_2_B_9_H_10_)^−^]. The copper atom in **3a** is coordinated to the two nitrogen atoms of the diimine, one phosphorus atom of the *nido*-diphosphane, and an oxygen atom resulting from the oxidation of the diphosphane. The bond distances for the oxidized diphosphane are Cu1–O1 2.165(4) Å and Cu1–P1 2.1665(13) Å, with the latter shorter than the Cu–P distances found in complex **1**. Cu–P distances in other [Cu(N^N)(dppnc)] complexes are also shorter than those found in **1**, which points out to a stronger Cu–P bond with *nido*-carborane diphosphanes, probably due to the additional electronic density in the carborane backbone, compared with that of the *closo*-carborane diphosphane. Cu–N distances in **1** may also be compared with that found in [Cu(N^N)(dppnc)], but a general trend is not so clear.

Further attempts to crystallize complex **2** in the absence of oxygen, under an argon atmosphere, or in a different solvent were unsuccessful. However, it was possible to obtain crystals of a different solvate of the oxidized compound under air. Analysis by X-ray studies of a crystal obtained by slow diffusion of hexane over a dichloromethane solution of **2** afforded the crystal structure of a different isomer of **3a** (**3b**), which crystallizes with dichloromethane as solvent. These results show that partial oxidation seems to be accessible for **2** and different isomers may be obtained. In isomer **3a**, the benzimidazole unit of the N^N ligand is oriented opposite to the open face of the carborane cage, whereas in **3b** the thiazole ring of the N^N ligand is oriented opposite to the open face of the carborane cage, as shown in Figure 2. Both isomers display very similar bond lengths and angles.

Oxidation and partial degradation of the *closo*-diphosphane through reaction with H_2_O_2_ has been reported.^32^ Oxidation of dppcc has been also observed in the synthesis of copper(II), nickel(II), and zinc(II) complexes during degradation process in refluxing ethanol;^33^ nevertheless, it has not been described during the synthesis and characterization of the copper complexes [Cu{(PR_2_)_2_C_2_B_9_H_10_}(PPh_3_)] (R = Ph, ^i^Pr), which were also carried out in refluxing ethanol,^31^ nor during the synthesis of the copper complexes [Cu(N^N)(dppnc)],^28,29^ analogous to **2**. These facts point to an oxidation of **2** during the crystallization process. We have carried out the corresponding studies in order to confirm this point. The ^31^P{^1^H} NMR spectrum of a solution of the crystals grown in acetone shows two main signals at 16.7 and 36.8 ppm, one at higher field corresponding to the lack of oxidized phosphorus atom and the other to the oxidized one. In addition, a weak signal at a 33.5 ppm appears, which could correspond to the compound with two oxidized phosphorus atoms (see SI). We also recorded the ^31^P{^1^H} NMR spectrum of **2** after months of its preparation, at low temperature, and two signals, at 13 and 17 ppm, are observed, which would correspond to two nonoxidized and no equivalent phosphorus atoms (see SI). Furthermore, we have also recorded the ^31^P{^1^H} NMR spectrum of a solution of **2** after 24 h of its preparation, and no oxidation product has been observed (see SI). These data prove that the emissive properties described below for **2**, not only in the solid state and as a film composite, but also in solution, correspond to the tetracoordinated derivative [Cu(N^N)(dppnc)] (**2**) and not to the isomers of **3**.

As oxidation of **2** seems to be accessible, but not complete, as **2** is still present in the reaction mixture, we tried a total transformation of **2** by bubbling pure oxygen through a solution of **2** in dichloromethane and some drops of hexane for one night. However, the ^31^P{^1^H} NMR spectrum shows that the result of the reaction is a mixture of **2** and the oxidized product **3** (probably a mixture of isomers) (see SI). Unsuitable crystals of **2** for X-ray measurements were obtained from these mixtures. Thus, synthesis of pure **3** or any of the isomers, as a pure product, is not easy and represents the subject of further work.

**Scheme 2:**
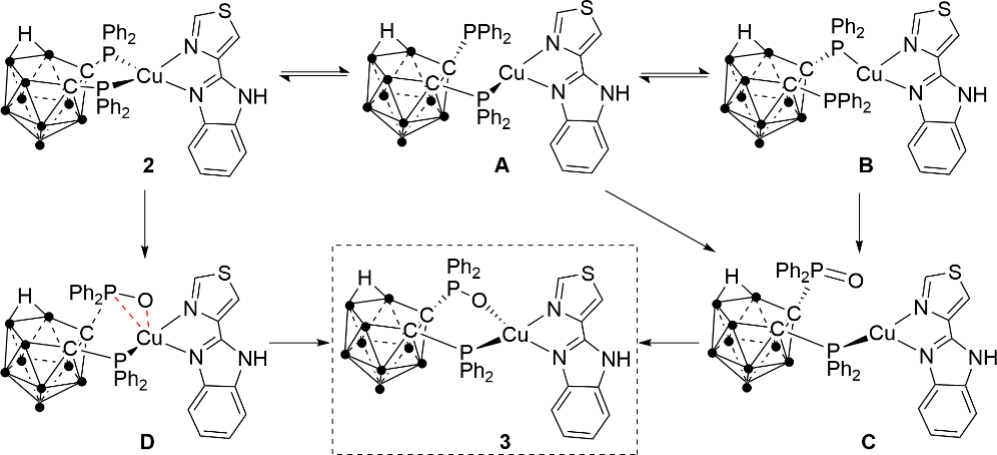
Formation of **3** from **2** Considering Cu–P Previous Dissociation or Not

From the data above, it seems that the presence of atmospheric oxygen is enough for the formation of the isomers of **3**, although only part of **2** is oxidized. We propose that the transformation of **2** in the oxidized **3** isomers may take place through a dissociative or associative pathway in solution, which are shown in Scheme 2, although we have not detected the corresponding intermedia. In the dissociative pathway, compound **2** would dissociate in solution to afford **A** or **B** (Scheme 2). The oxidation of the uncoordinated phosphorus atom could lead to intermediate **C** that can further coordinate to the copper center to give **3**.

Alternatively, the associative pathway would take place through the simultaneous formation of one oxygen copper bond and breaking of the Cu–P bond through **D**, leading to the formation of **3**. These processes are in equilibria from which mixtures of **2** and **3** are obtained.

This oxidation has not been detected for compound **1** bearing the analogous *closo*-carborane-diphosphane, nor has it been reported for three or four other coordinated copper(I) complexes with the anionic *nido*-carborane-diphosphane.

### Emission of Powder Samples

Powder samples of complexes **1** and **2** are luminescent upon excitation at about 400 nm (Table 1 and SI). The emissive behavior of complex **1** in the solid state (powder) shows a strong thermochromism. At room temperature, a band is observed in the red region, whereas the emission is blue-shifted at 77 K, and a green-yellow emission is observed. Rigidochromic effects have been claimed as responsible for the blue shift of emissions upon cooling.^25^ The opposite trend is observed for compound **2**, which displays emission in the green-yellow region showing a small red shift (11 nm) upon cooling. An increment of the lifetime is observed for both complexes upon cooling. This increment is much higher for **2** (from 25 to 2900 μs).

**Table 1:** Emissive Properties of Complexes **1** and **2**

conditions	λ_em_^b^	λ_ex_^b^	Φ^c^	τ^d^
Compound **1**				
film	540	360, 395(sh)	<1	
powder, RT	700	450	<1	41.5 (0.991)
powder, 77 K	542	400		336.8 (0.982)
frozen soln^a^	450	270		1044.9 (0.999)
Compound **2**				
film	542	395	10	23.0 (0.998)
powder, RT	547	455	16	25.8 (0.999)
powder, 77 K	558	335, 423		2932.3 (0.996)
frozen soln^a^	530	315, 395		3885.5 (0.998)

a 10^–3^ M acetone solution.

b λ_em_, emission maxima; λ_ex_, excitation maxima (in nm). sh = shoulder.

c In %; λ_ex_ = 395 nm.

d In μs (χ^2^). The studies have been carried out using the λ_em_ and λ_ex_ maxima.

**Figure 3:**
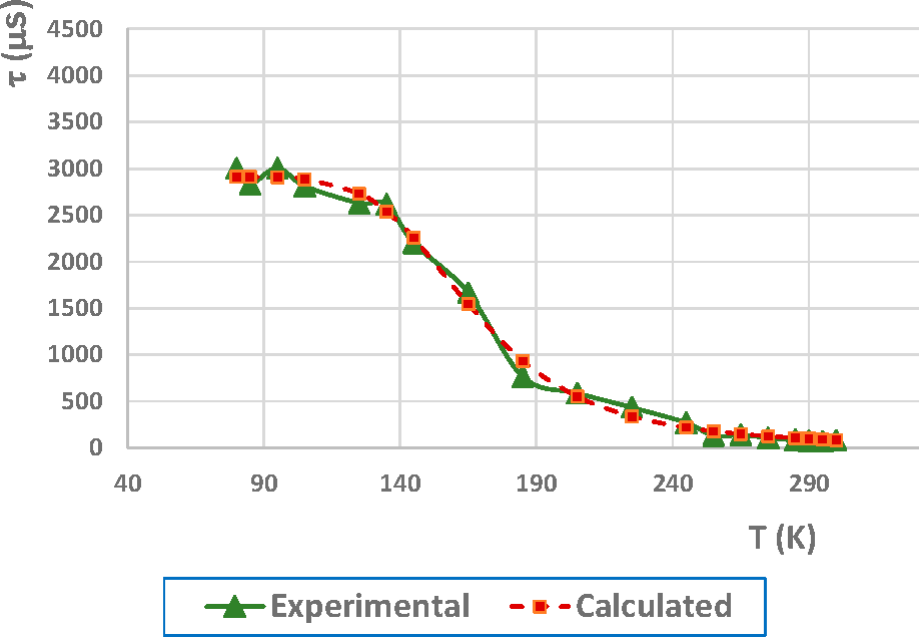
Temperature dependence of the emission lifetime of complex **2** in the solid state with fitting values using eq 1.

The shift to lower energies of the emission maximum and the increment of the lifetime upon cooling point to TADF behavior for **2**. Thus, we have studied lifetime vs temperature for **2** with the aim of analyzing TADF behavior. By fitting the observed τ at different temperatures to eq 1 (*K*_B_ = Boltzmann constant) using the least-squares fitting method (Figure 3), the calculated values are τ(S1) = 0.34 μs, τ(T1) = 2.92 ms, and Δ*E*(S_1_ – T_1_) = 925 cm^–1^.
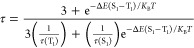
1

Crystals of **3** are brightly emissive. As discussed above, further efforts are needed in order to obtain isomers of **3** as pure samples which would allow characterization of their emissive properties.

### Emission of PMMA Films

As mentioned above, we have checked by NMR that the compound used for luminescence studies, not only in the solid state, but also as film composite and in solution, corresponds to the tetracoordinated derivative [Cu(N^N)(dppnc)] (**2**) and not to isomers of **3**, as no signal corresponding to the presence of **3** is observed in the ^31^P{^1^H} NMR spectrum of a sample of **2** after months of its preparation. During frozen solution measurements and film preparation, formation of **3** is also discarded, because no signal corresponding to the presence of **3** is observed in the ^31^P{^1^H} NMR spectrum of a solution of **2** after more than 1 day from the preparation of the solution, which represents a longer period from that employed for luminescence measurements and film preparation.

**Figure 4:**
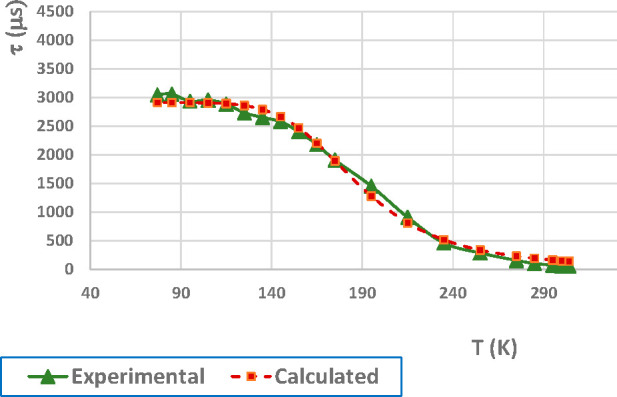
Temperature dependence of the emission lifetime of PMMA film at 5 wt % of complex **2** with fitting values using eq 1.

Composites of both complexes as PMMA films at 5 wt % lead to emissions in the green region. Representation of the lifetime at different temperatures and fitting to eq 1 for the composite of compound **2** leads to Δ*E*(S_1_ – T_1_) = 1027 cm^–1^, τ(S_1_) = 0.39 μs, and τ(T_1_) = 2.91 ms (Figure 4). The powder value for the Δ*E*(S_1_ – T_1_) gap is a bit smaller than in the less rigid PMMA film but does not show a significant difference.

**Figure 5:**
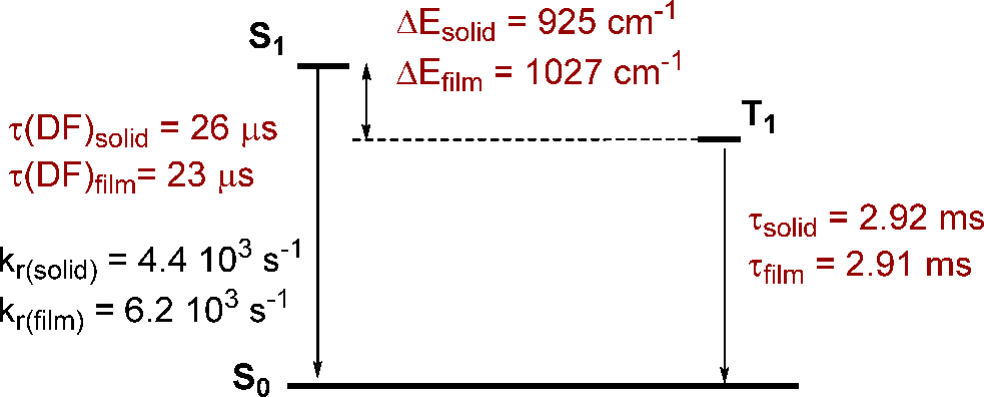
Energy level diagram for **2** showing data calculated from eq 1 as well as τ(DF) (thermal activated delayed fluorescence lifetime) and *k*_r_ (radiative constant) values.

The results are summarized in Figure 5. Prompt fluorescence is not observed as the ISC process is very fast (ps).^19^ Δ*E*(S_1_ – T_1_) values for **2** are in the range considered for practical use of TADF^19^ and may be compared with those calculated for similar [Cu(N^N)(dppnc)] compounds using the same method, which range from 741 to 1195 cm^–1^.^28^ We can conclude that the change of a phenanthroline diimine ligand by the N^N ligand does not lead to a significant decrease in the Δ*E*(S_1_ – T_1_) gap.

Quantum yields of powder and PMMA films at 5 wt % of complex **2** are 16% and 10%, respectively, whereas those for complex **1** are about 1%. Values observed for **2** are in between those reported for [Cu(N^N)(dppnc)] complexes,^19,20^ which range between ca. 5% and 32% in film and 4% and 39% in powder samples. After removing the excitation light, from the emission spectra, the CIE 1931 coordinates have been obtained (SI).

A noticeable point is the different emission energy observed at room temperature for the composite and powder samples of **1**. Powder samples (red emissive) display an important blue shift compared with the PMMA composites at 5 wt % (weakly yellow emissive).

### Emission in Frozen Solutions

UV–vis spectra have been recorded for complexes **1** and **2**, as well as for the ligands N^N and dppcc (see SI). The diphosphane dppcc displays an intense band at 273 nm. Intense bands at about 300 nm and a shoulder at about 310 nm are observed for the N^N ligand as well as for complexes **1** and **2**. In addition to this intense band, compound **1** displays a shoulder at 347 nm and a weak band centered at 393 nm. Thus, absorption of compound **2** resembles that of the N^N ligand, whereas that of **1** displays additional bands, probably related to charge transfer transitions.

No emission has been observed for **1** and **2** in 10^–3^ M acetone or dichloromethane solutions at room temperature.

**Figure 6:**
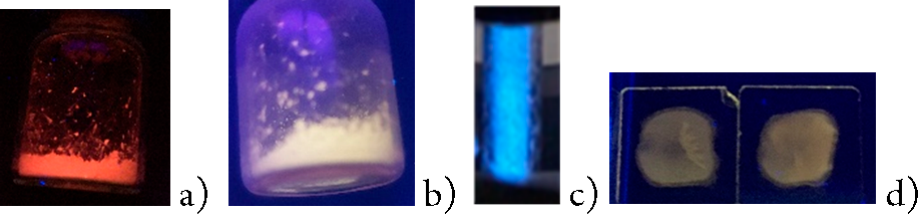
Compound **1** under UV (360 nm) light in the solid state at room temperature (a) and 77 K (b), as a frozen acetone solution (c), and in PMMA film (d).

**Figure 7:**
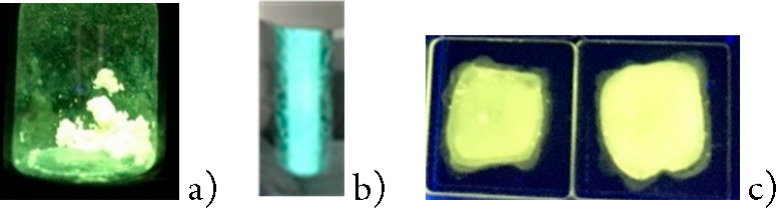
Compound **2** under UV (360 nm) light in the solid state at room temperature (a), as a frozen acetone solution (b), and in PMMA film (c).

Frozen acetone glasses are highly emissive. Their maxima are blue-shifted, compared with the values for powder samples of PMMA composites at 5 wt % and display blue (**1**) or green (**2**) emission. Figures 6 and 7 summarize the emissive color of complexes **1** or **2**, respectively, depending on the aggregation state.

These data reveal that the emissive behavior of the cationic tetracoordinated complex **1** is highly dependent on temperature and aggregation state. Compound **1** follows the expected rule as a blue shift of the emission is observed upon cooling the solid powder. Different explanations for the blue shift upon cooling in the same media have been attributed to rigidochromism,^34^ but also to emissions from different excited states. These excited states exhibit different distortions from the ideal tetrahedral geometry.^35^ The explanation for the modification of the emission energy with the media is more complicated. Some literature studies attribute the red shift seen when changing from the solid powder to the film to an expected structural relaxation,^26^ but this red shift, as observed for compound **1** and other complexes reported in the literature, may not be considered a general trend.^19^ Thus, other factors may be important.

## Theoretical Studies

In order to get insight into the different emissive behaviors of **1** and **2**, TD-DFT calculations were carried out for both complexes. The X-ray atomic coordinates were used in the case of compound **1** as a starting point. Comparisons of the copper environment in the crystal structure and optimized geometry are shown in Table 2. Longer Cu–P, but shorter Cu–N, distances are observed in the optimized geometry. Chelate angles are diminished in the optimized structure. As a result, distortion from the tetrahedral geometry is increased in the optimized structure, as may be shown by the α angle.

**Table 2:** Selection of Bond Distances (Å) and Angles (°) in **1** and **2**

	Cu–P	Cu–N	N–Cu–N	P–Cu–P	α^a^
Compound **1**					
X-ray data	2.2398	2.073	81.60	98.18	82.70
	2.2242	2.057			
optimized	2.3548	2.236	78.76	95.88	75.84
	2.3528	2.105			
Compound **2**					
optimized	2.357	2.216	78.10	90.57	78.02
	2.326	2.139			

a α = angle between N–Cu–N and P–Cu–P planes.

**Figure 8:**
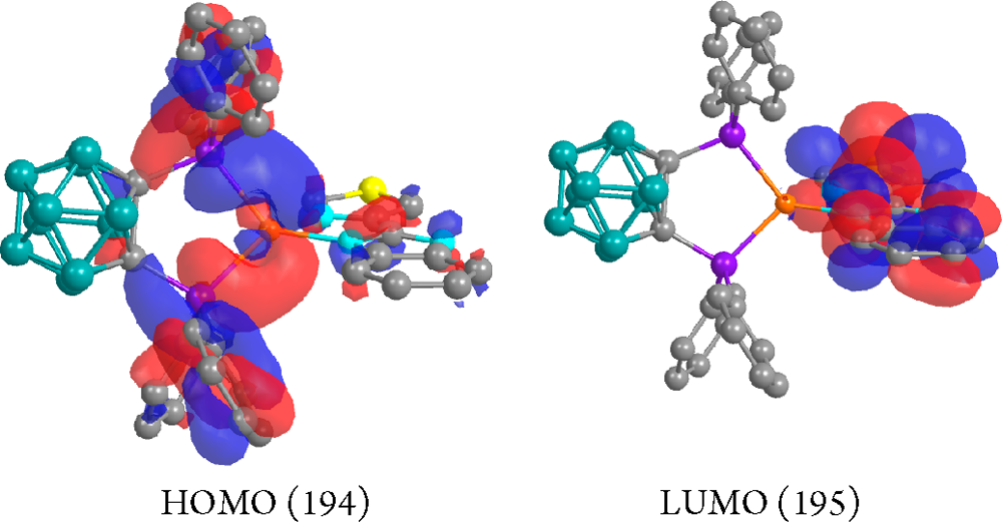
HOMO and LUMO calculated molecular orbitals for compound **1**.

At the ground-state geometry, the excitation energies were calculated, being the only contribution to the S_0_ → S_1_ transition (Table 3) corresponding to the HOMO–LUMO orbitals. The HOMO (Figure 8) is built mostly by contributions of the copper atom (32%), the phosphorus (30%), and phenyl rings (22%) of the carborane diphosphane ligand (simple contributions), with an almost negligible contribution of the N^N ligand.

**Table 3:** Calculated Transitions for **1** and **2**

	λ_ex_^a^	Transition (f)		Orbitals	λ^b^
**1**	450	S_0_ → S_1_	(0.006)	194 (HOMO) → 195 (LUMO)	409
		S_0_ → T_1_		194 (HOMO) → 195 (LUMO)	451
				193 (HOMO–1) → 195 (LUMO)	
**2**	455	S_0_ → S_1_	(0.0047)	191 (HOMO–1) → 193 (LUMO)	614
				192 (HOMO) → 193 (LUMO)	
		S_0_ → S_2_	(0.0017)	191 (HOMO – 1) → 193 (LUMO)	532
				192 (HOMO) → 193 (LUMO)	
		S_0_ → S_3_	(0.0420)	191 (HOMO–1) → 194 (LUMO+1)	438
				192 (HOMO) → 194 (LUMO+1)	
		S_0_ → T_1_		191 (HOMO–1) → 193 (LUMO)	631
				192 (HOMO) → 193 (LUMO	

a λ_ex_ = excitation maximum in the solid state (nm).

b λ = calculated values (nm).

In the LUMO (Figure 8), all of the electronic density is distributed thought the N^N ligand. Thus, the origin of the absorption leading to the emission seems to be a metal–ligand(diphosphane) (ML) to the N^N ligand (L′) charge transfer transition (MLL′CT). HOMO to LUMO and HOMO–1 to LUMO contribute to the S_0_ → T_1_ transition (Table 3).

**Figure 9:**
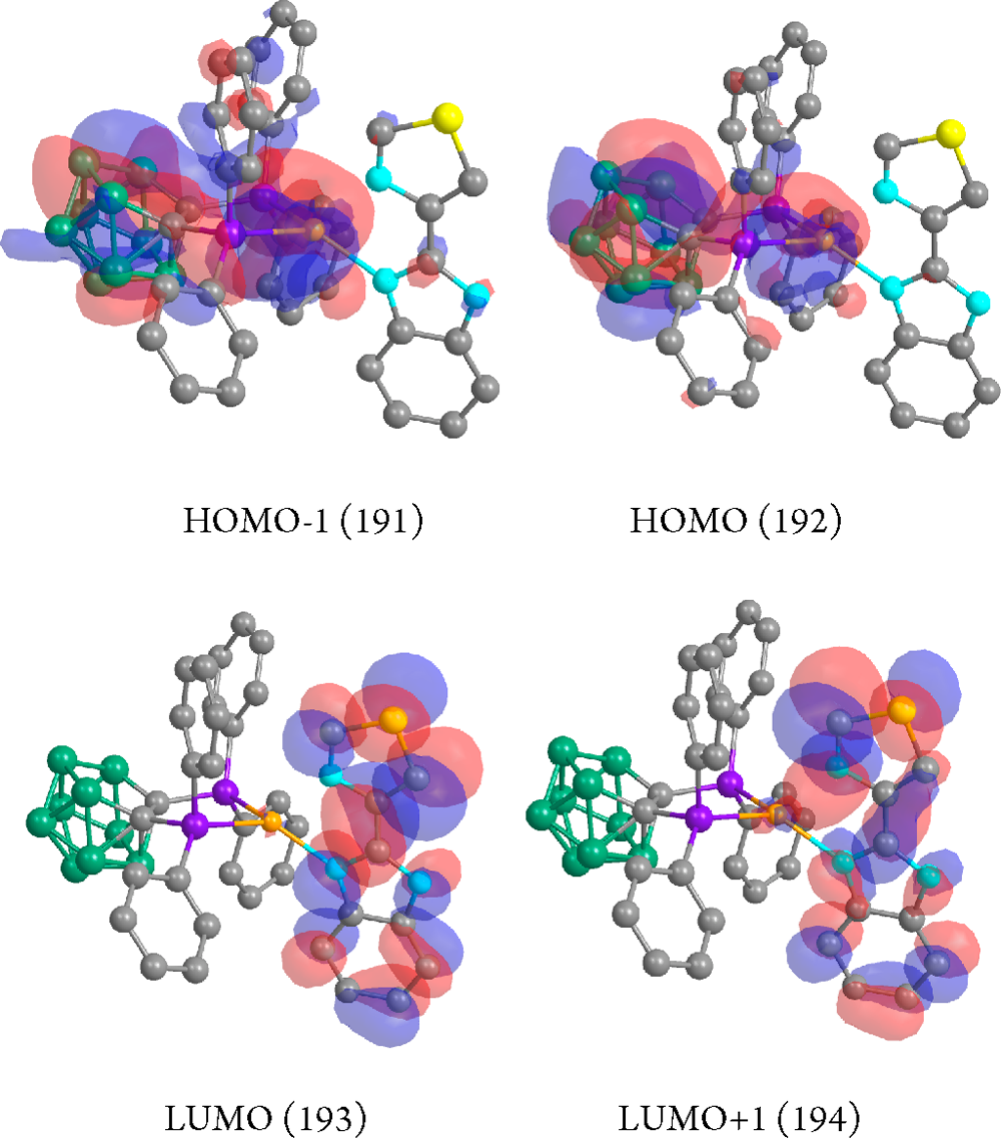
HOMO–1, HOMO, LUMO, and LUMO+1 calculated molecular orbitals for compound **2**.

Geometry parameters for the optimized structure of **2** are shown in Table 2. For compound **2**, transitions S_0_ → S*_n_* (*n* = 1–3, Table 3) involve HOMO–1, HOMO, LUMO, and LUMO+1 at the ground-state geometry. The electronic density of both HOMO and HOMO–1 (Figure 9) is mainly located in the copper center and carborane backbone with an additional contribution of the phosphorus of the *nido*-diphosphane. The contribution of the copper atom represents 22% and 9% in HOMO–1 and HOMO, respectively. That of the carborane backbone represents 33% and 60% in HOMO–1 and HOMO, respectively, and that of the phosphorus atoms of the diphosphane 26% and 14% in HOMO–1 and HOMO, respectively. The LUMO and LUMO+1 are mainly located in the N^N ligand (Figure 8).

Thus, the electronic transition may also be described as metal–ligand(diphosphane) (ML) to the N^N ligand (L′) charge transfer transition (MLL′CT), but important differences, when compared with compound **1**, are found related to (i) the contribution of the *nido*-carborane skeleton, negligible in **1**, (ii) the lesser contribution of the copper atom found for **2**, and (iii) contribution of the phenyl rings of the carborane diphosphane found in **1**, negligible in **2**. HOMO to LUMO and HOMO–1 to LUMO contribute to the S_0_ → T_1_ transition (Table 3).

Theoretical studies reported on other [Cu(N^N)(dppnc)] complexes [N^∧^N = neocuproine, 1,10-phenanthroline, or functionalized neocuproine],^28,29^ for which TADF has been proposed, have attributed the origin of the emissions to MLCT or MLCT mixed with LL′CT transitions. These studies have revealed no contribution or negligible representation of the carborane skeleton of the dppnc ligand in the orbitals mainly responsible of the excitation transitions leading to emission. In all these complexes with the *nido*-diphosphane, the dppnc^–^ contribution of the phenyl rings of the diphosphane^28,29^ seems to be less important than in **1** with the *closo*-diphosphane dppcc, for which TADF has not been found. Further work is needed in order to extend this effect as a general rule when comparing similar complexes with the *closo* (dppcc) and *nido* (dppnc^–^) diphosphanes.

## Conclusion

The presence of an anionic *nido*-diphosphane (dppnc^–^) or the neutral *closo*-diphosphane (dppcc) in the tetracoordinated [Cu(N^N)(P^P)]*^n^* (*n* = 0, 1) complexes represents the key factor for the tuning of the emissive properties. The neutral compound [Cu(N^N)(dppnc)] exhibits TADF and singlet and triplet lifetimes; in addition, the Δ*E*(S_1_ – T_1_) is almost unmodified from the solid to PMMA composites at 5% wt. The cationic complex [Cu(N^N)(dppcc)]PF_6_ exhibits a strong thermochromic behavior. Its emissive excited state is highly dependent on the medium and temperature, displaying emissions which span from the red (solid at room temperature) to yellow (solid at 77 K) and blue (frozen acetone solution). It does not exhibit TADF. Charge transfer (copper to diimine) transitions have been claimed as responsible for the emissive behavior of many complexes of stoichiometry [Cu(N^N)(P^P)]^0/+^. More specifically, for different complexes with phenanthroline type N^∧^N ligands and *nido-*carborane diphosphanes, theoretical calculations lead to such a conclusion (in some cases with some mixed ligand to ligand charge transfer (LLCT) character).^28,29^ Previously reported studies for similar complexes with the *nido*-diphosphane may conclude that changes in the diimine lead to different emission energies and quantum yields. In addition, steric effects avoiding distortion in the excited state have been claimed as very relevant in the emissive behavior of [Cu(N^∧^N)(P^∧^P)]^0/+^ compounds and, thus, in the design of TADF materials. Complexes **1** and **2** do not show significant differences in their steric hindrance, with this being the unique modification that is related to the partial degradation of the carborane cage. These results point to electronic effects as another relevant factor in controlling their luminescence behavior and more specifically TADF emission. For complexes **1** and **2**, the nature of the diphosphane represents the unique difference. The electron-withdrawing effect of carborane is supposed to be diminished in the mononegative *nido*-carborane-diphosphane, compared with the neutral *closo*-carborane-diphosphane. Deeper understanding of the relevance of the concrete effect of these electronic differences between the *closo* and *nido* clusters is needed, but theoretical studies on **1** and **2** reveal that MLL′CT transitions are responsible for the transitions related to the excitations leading to luminescence with the main differences regarding a lesser contribution of the copper center in compound **2** with the *nido*-diphosphane; the contribution of the diphosphane skeleton (carborane cage) found in **2** is not relevant in **1** with the *closo*-diphosphane, and the important contribution of the orbitals of the phenyl rings of the diphosphane is found in **1**, which is not present or is negligible in **2**, as in other previously reported complexes with the *nido*-diphosphane.

## Experimental Section

### Instrumentation

NMR spectra were carried out in a Bruker AV 400 or 300 in CDCl_3_ if solvent is not specified, and chemical shifts (ppm) are reported relative to the solvent peaks of the deuterated solvent.^36^

Steady-state photoluminescence spectra were recorded with a Jobin-Yvon Horiba Fluorolog FL-3-11. Lifetime measurements were recorded with a Fluoromax phosphorimeter accessory containing a UV xenon pulsed flash tube. Multichannel scaling (MSC) has been used as the measuring method. Data were fitted to monoexponential functions, and the corresponding fitting curves are included in the SI. An OptistatDN Oxford variable temperature liquid nitrogen cryostat has been used for lifetime studies at different temperatures and a liquid nitrogen dewar assembly for steady-state studies at 77 K. Quantum yields were measured by the absolute method using a Hamamatsu Quantaurus-QY C11347 compact one-box absolute quantum yield measurement system. Quartz tubes were used for the measurement of solid samples. For film preparation, ca. 4 mg of compound and ca. 76 mg of PMMA were dissolved in 1 mL of CH_2_Cl_2_ in order to obtain films which contain about at 5 wt % of the copper compound. The mixture was sonicated for 15–20 min. Films were prepared by drop casting the resulting solution. Two or three films of each complex were prepared as well as two or three reference samples. Each sample was measured using each reference in order to prove the reproducibility of the results.

### Crystallography

Crystals suitable for X-ray studies were obtained by diffusion of *n*-hexane over a solution of the corresponding compound in dichloromethane (**1**, **3b**) or acetone (**3a**). Crystals were mounted in inert oil on a glass fiber and transferred to the cold gas stream of a SMART APEX (**1**, **3a**) or mounted on a MiTeGen Crystal micromount and transferred to the cold gas stream of a Bruker D8 VENTURE (**3b**) diffractometer. Data were collected using monochromated Mo Kα radiation (λ = 0.71073 Å), with scan type ω. Absorption corrections based on multiple scans were applied with the program SADABS;^37^ for **3b**, numerical absorption corrections based on crystal face indexing have been performed. The structures were refined on *F*^2^ using the program SHELXL-2018.^38^ All non-hydrogen atoms were refined anisotropically. Hydrogen atoms were included using a riding model. CCDC depositions 2076381 (**1**), 2076380 (**3a**), and 2088767 (**3b**) contain the supplementary crystallographic data. These data can be obtained free of charge by The Cambridge Crystallography Data Center.

### Theoretical Studies

The Gaussian 09 program was used in order to carry out the DTF calculations. Geometry optimizations were performed on the ground state using the hybrid B3LYP functional and the basis sets def2-SVP (for C, S, and H atoms),^7^ def2-TZVP (for P, N, and O atoms), and LANL2DZ for the copper atom.^28,29^ For the copper atom, the corresponding associated pseudopotential to LANL2DZ was applied.

### Synthesis

The synthetic procedures were carried out under an Ar atmosphere, using Schlenk techniques. Dry degassed solvents were used. The starting materials [Cu(CH_3_CN)_4_]PF_6_ and N^N [2-(4-thiazolyl)benzimidazole] are commercially available and were used as received. The diphosphane 1,2-(PPh_2_)_2_-1,2-C_2_B_10_H_10_ was prepared using a published procedure.^39^

#### Synthesis of [Cu(N^N)(dppnc)]PF_6_ (**1**)

To a solution of [Cu(CH_3_CN)_4_](PF_6_) (0.1 mmol, 37.2 mg) in dichloromethane was added [1,2-(PPh_2_)_2_-1,2-C_2_B_10_H_10_] (0.1 mmol, 51.3 mg). The mixture was stirred for 30 min, and 2-(4-thiazolyl)benzimidazole (0.1 mmol, 20.8 mg) was added. The mixture was stirred for 1 h. Then, it was evaporated to a minimum volume. Addition of *n*-hexane led to the precipitation of **1** as a pale yellow solid, which was filtered and dried under vacuum. Yield: 87.2 mg, 94.5%. Q-TOF: *m*/*z* 777 (34.3%) [M – PF_6_]. ^1^H NMR (400 MHz, acetone-*d*_6_, 25 °C): δ = 12.95 (s, 1H), 9.58 (s, 1H), 8.87 (s, 1H), 7.96–7.72 (m, 10H), 7.70–7.39 (m, 14H), 3–0.6 (m, 10H) ppm. ^31^P{^1^H} NMR (121 MHz, acetone-*d*_6_, 25 °C): δ = 13.5 ppm.

#### Synthesis of [Cu(N^N)(dppnc)] (**2**)

To a solution of [Cu(CH_3_CN)_4_](PF_6_) (0.1 mmol, 37.2 mg) in ethanol was added 1,2-(PPh_2_)_2_-1,2-C_2_B_10_H_10_ (0.1 mmol, 51.3 mg). The mixture was stirred for 30 min, and 2-(4-thiazolyl)benzimidazole (0.1 mmol, 20.8 mg) was added. The mixture was refluxed for 1 h. Concentration of the solution led to precipitation of a solid which was filtered and dried under vacuum. Yield: 52.9 mg, 69%. Q-TOF: *m*/*z* 789.22 (5.4%) [M + Na]. ^1^H NMR (400 MHz, acetone-*d*_6_, 25 °C): δ = 12.95 (s, 1H), 8.58 (s, 1H), 7.91–7.09 (m, 20H, Ph; 5H, N^N), 3–0.6 (m, 10H), −1.85 (s, br, 1H) ppm. ^31^P{^1^H} NMR (121 MHz, acetone-*d*_6_, 25 °C): δ = 16.6 ppm.

## Supporting Information Available

Additional details of the emissive properties and theoretical studies (PDF)
Coordinates of the optimized structures (XYZ)

## Supplementary Material

ic1c03092_si_001.pdf

ic1c03092_si_003.xyz

## Accession Codes

CCDC 2076380–2076381 and 2088767 contain the supplementary crystallographic data for this paper. These data can be obtained free of charge via www.ccdc.cam.ac.uk/data_request/cif, or by emailing data_request@ccdc.cam.ac.uk, or by contacting The Cambridge Crystallographic Data Centre, 12 Union Road, Cambridge CB2 1EZ, UK; fax: +44 1223 336033.
