# Correction to “*closo*- or *nido*-Carborane Diphosphane as Responsible for Strong Thermochromism or Time Activated Delayed Fluorescence (TADF) in [Cu(N^N)(P^P)]^0/+^”

**DOI:** 10.1021/acs.inorgchem.2c01209

**Published:** 2022-05-13

**Authors:** Adrián Alconchel, Olga Crespo, Pilar García-Orduña, M. Concepción Gimeno

The authors regret that “time activated delayed fluorescence (TADF)” should be “thermally activated delayed fluorescence” in the title, abstract, and introduction of the manuscript.

