# Supplementary material for: Closo- or Nido-Carborane Diphosphane as Responsible for Strong Thermochromism or Time Activated Delayed Fluorescence (TADF) in [Cu(N^N)(P^P)]0/+
Source: Inorg Chem. 2021 Nov 23;60(23):18521–8. doi: 10.1021/acs.inorgchem.1c03092 (PMC8653344; doi:10.1021/acs.inorgchem.1c03092)
Supplement: Supplementary file 1 — ic1c03092_si_001.pdf [file ic1c03092_si_001.pdf]

ELECTRONIC SUPPLEMENTARY INFORMATION

Closo- or *Nido-*Carborane Diphosphane as Responsible for Strong Thermochromism or TADF in $[Cu(N^\wedge N)(P^\wedge P)]^{0/+}$

Adrián Alconchel, Olga Crespo,* Pilar García-Orduña, M. Concepción Gimeno*

Departamento de Química Inorgánica, Instituto de Síntesis Química y Catálisis Homogénea (ISQCH). Universidad de Zaragoza-CSIC. E-50009 Zaragoza, Spain.

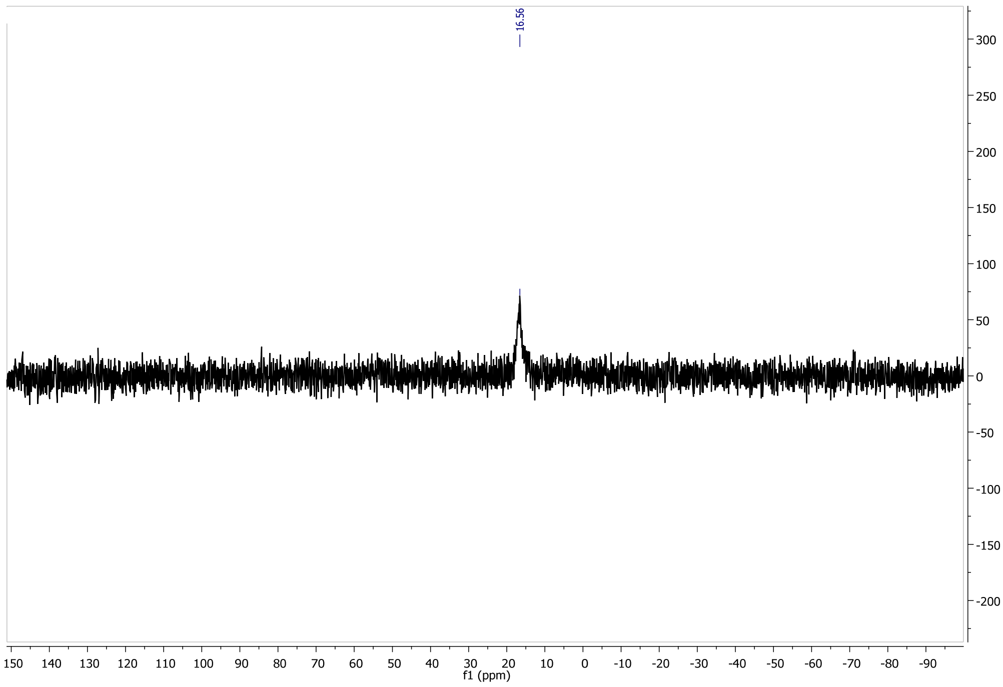

Figure S1. $^{31}P\{^1H\}$ NMR spectrum of 2 in d6-acetone at room temperature

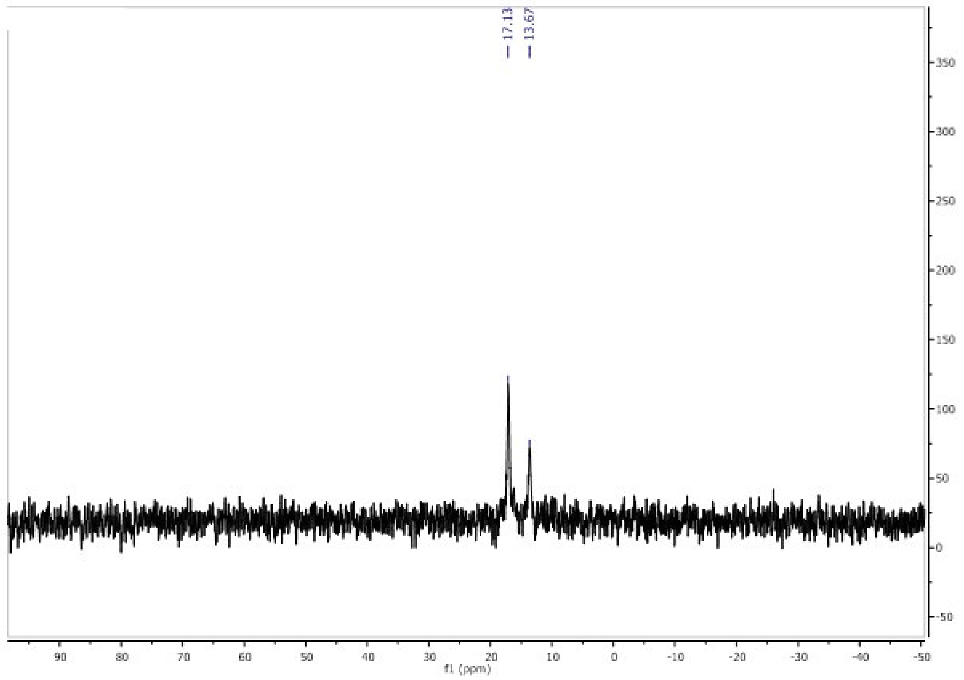

Figure S2. $^{31}P\{^1H\}$ NMR spectrum of 2 in d6-acetone at 77 K

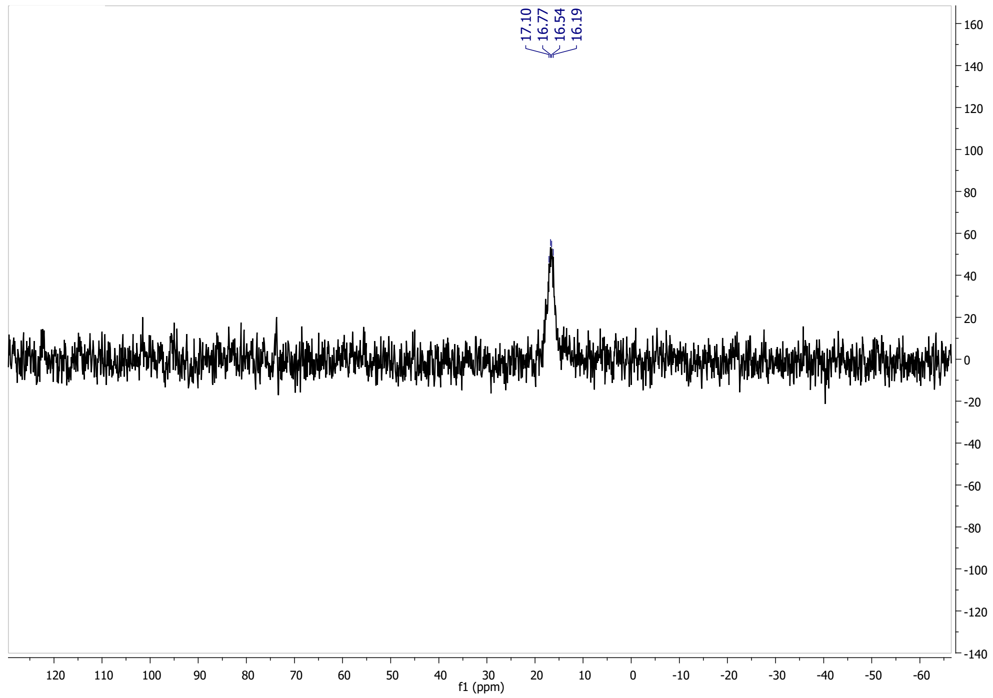

Figure S3. $^{31}P\{^1H\}$ NMR spectrum of 2 in d6-acetone. The same solution used for recording the spectrum in acetone at 77 K (Figure S2) after one day.

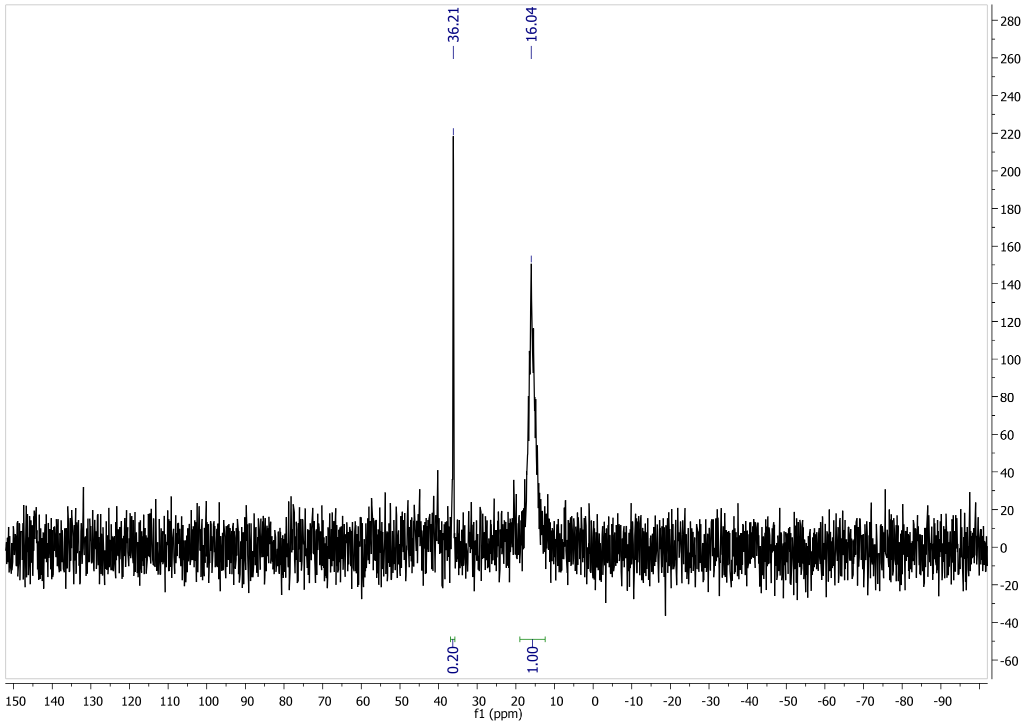

Figure S4. $^{31}P\{^{1}H\}$ **NMR, in d6-acetone, of the solid obtained after concentration of a solution of 2 in dichloromethane and some drops of n-hexane, through which pure oxygen has been bubbling for one night.**

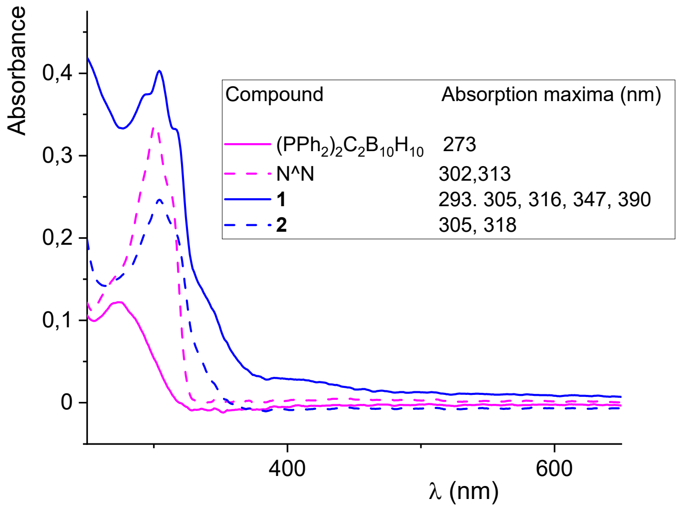

Figure S5. UV-Vis spectra of the two ligands and complexes 1 and 2 in dichloromethane solution 10^{-5} M at room temperature

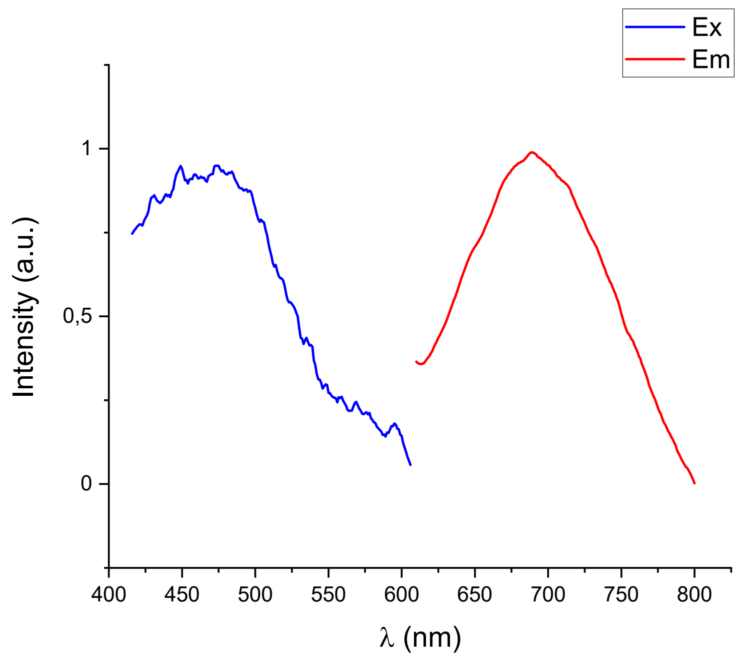

Figure S6. Emission and excitation spectra of 1 in solid state at room temperature. Emission spectrum with excitation at 290 nm. Excitation spectrum with emission at 730 nm. Intensity of the excitation at 290 is much lower than that at 450 nm (shown in the Figure), both leading to the red emission. Excitation at 450 led to worse spectrum, probably due to the presence or artefacts, the emission presented is that upon excitation at 290 nm).

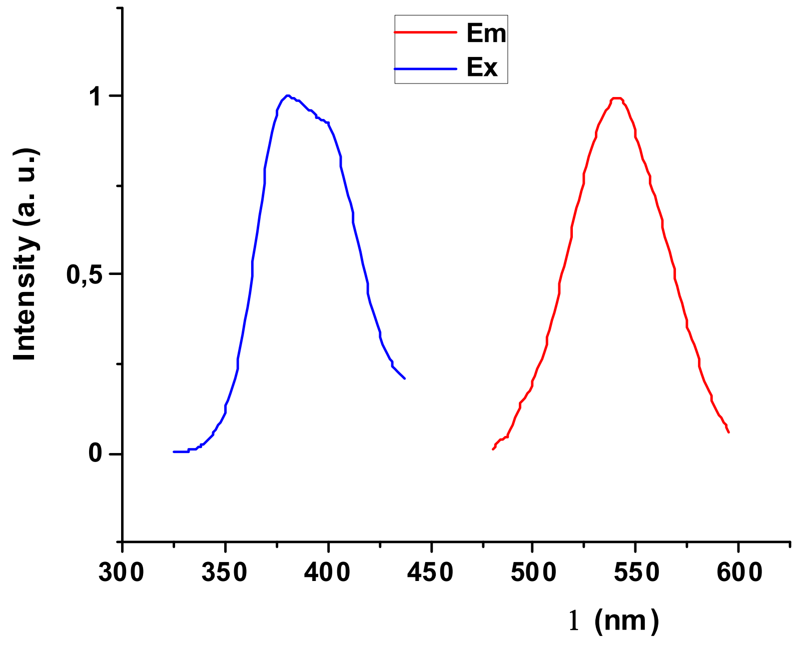

Figure S7. Emission and excitation spectra of 1 in solid state at 77 K. Emission spectrum with excitation at 390 nm, excitation spectrum with emission at 550 nm.

zz

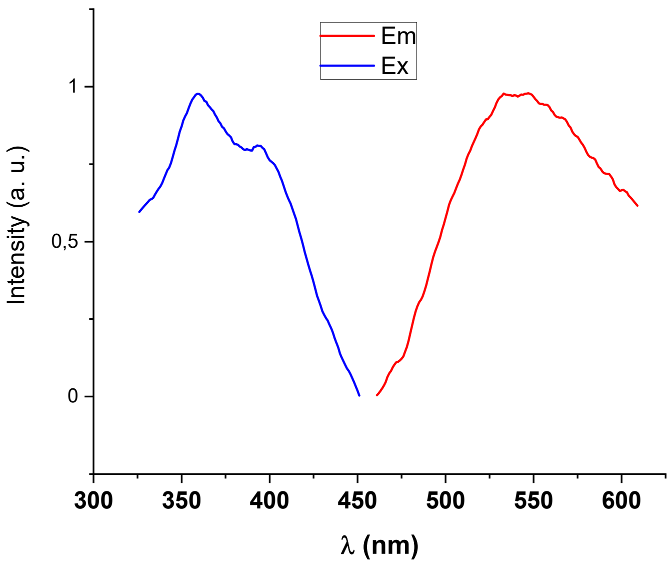

Figure S8. Emission and excitation spectra of 1 in PMMA film at 5% wt. Emission spectrum with excitation at 390 nm, excitation spectrum with emission at 540 nm.

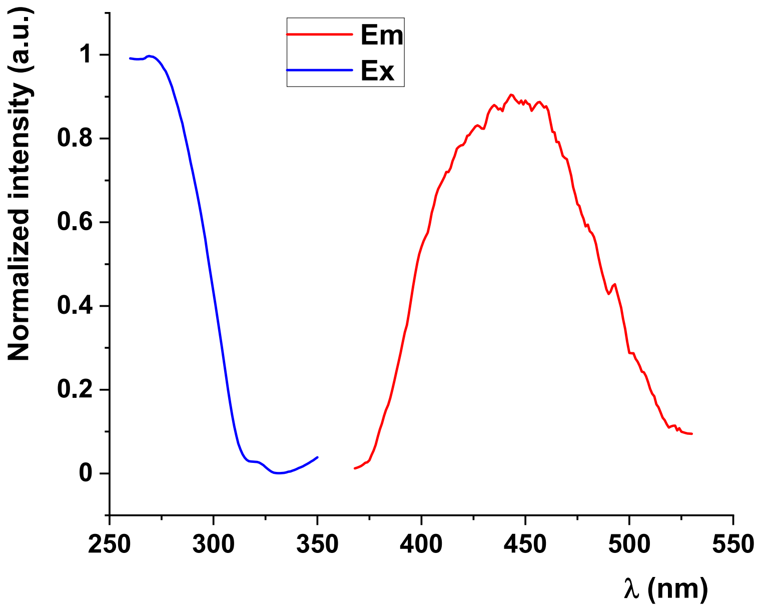

Figure S9. Emission and excitation spectra of 1 in 10^{-3} M acetone solution at 77K. Emission spectrum with excitation at 300 nm, excitation spectrum with emission at 400 nm.

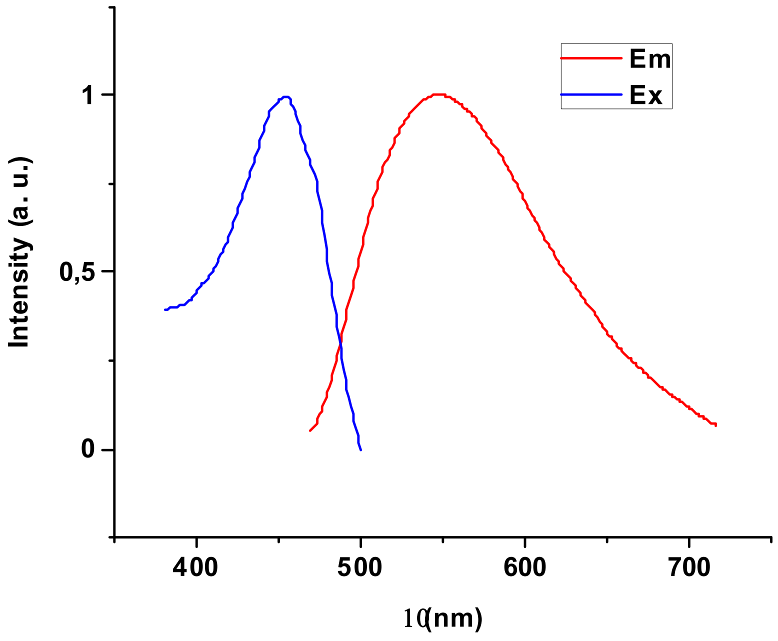

Figure S10. Emission and excitation spectra of 2 in solid state at room temperature. Emission spectrum with excitation at 420 nm, excitation spectrum with emission at 542 nm.

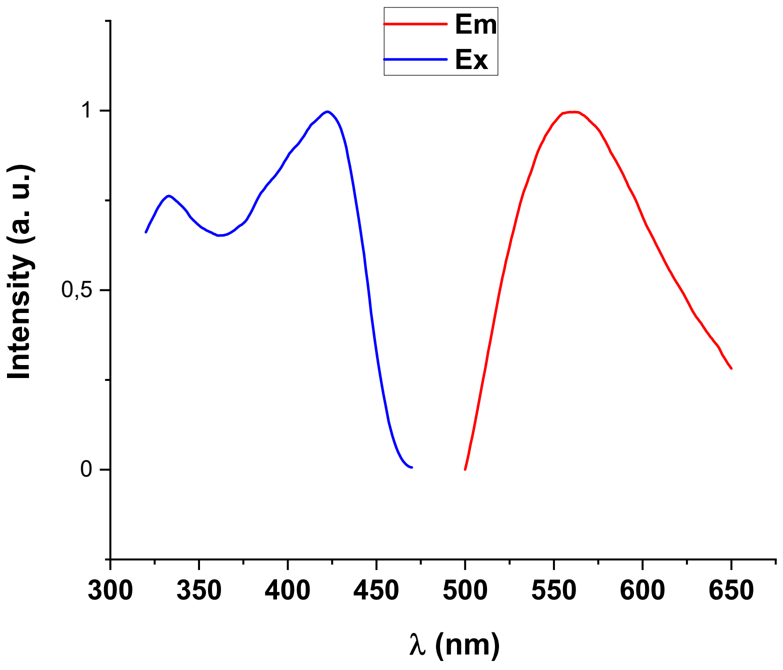

Figure S11. Emission and excitation spectra of 2 in solid state at 77 K. Emission spectrum with excitation at 423 nm, excitation spectrum with emission at 558 nm.

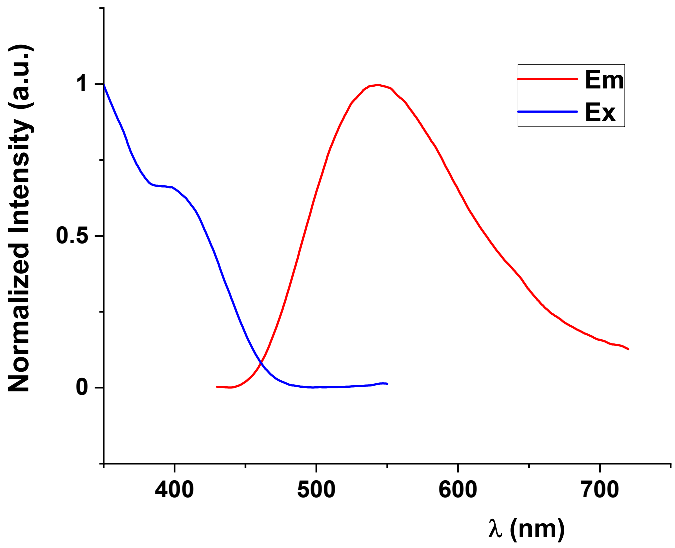

Figure S12. Emission and excitation spectra of 2 in PMMA film at 5% wt. Emission spectrum with excitation at 390 nm, excitation spectrum with emission at 542 nm.

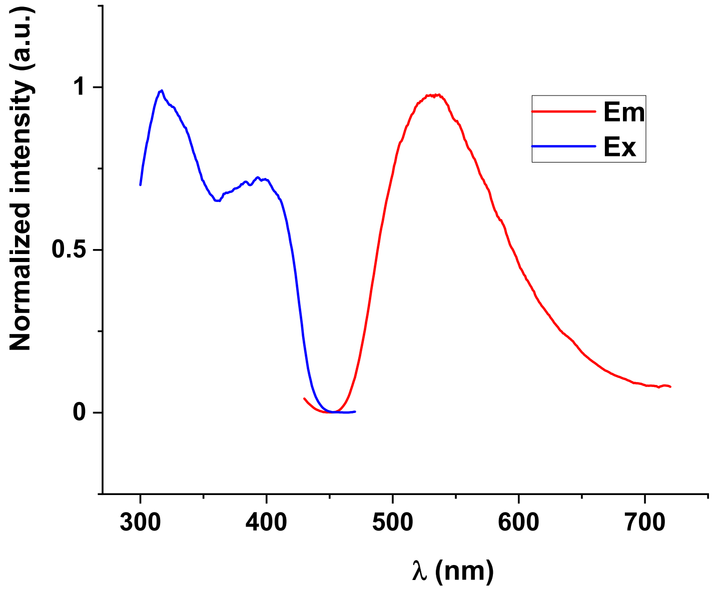

Figure S13. Emission and excitation spectra of 2 in 10^{-3} M acetone solution at 77K. Emission spectrum with excitation at 390 nm, excitation spectrum with emission at 530 nm.

	y0	y0	A1	A1	t1	t1	Statistics	Statistics
	Value	Standard E	Value	Standard E	Value	Standard E	Reduced C	Adj. R-Squ
S1	27,64517	1,0962	866,16686	24,6831	0,0415	0,00201	54,45722	0,99107

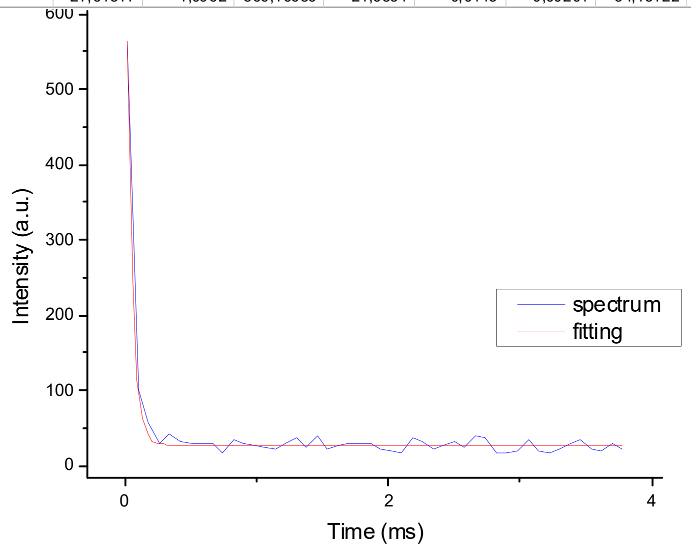

Figure S14. Decay curve and fitting data for complex 1 in the solid state at room temperature (λex = 400 nm; λem = 710 nm)

	y0	y0	A1	A1	t1	t1	Statistics	Statistics
	Value	Standard Er	Value	Standard Er	Value	Standard Er	Reduced C	Adj. R-Squ
S1	319,81718	2,76255	2602,4269	23,67556	0,3368	0,00412	2757,1143	0,98223

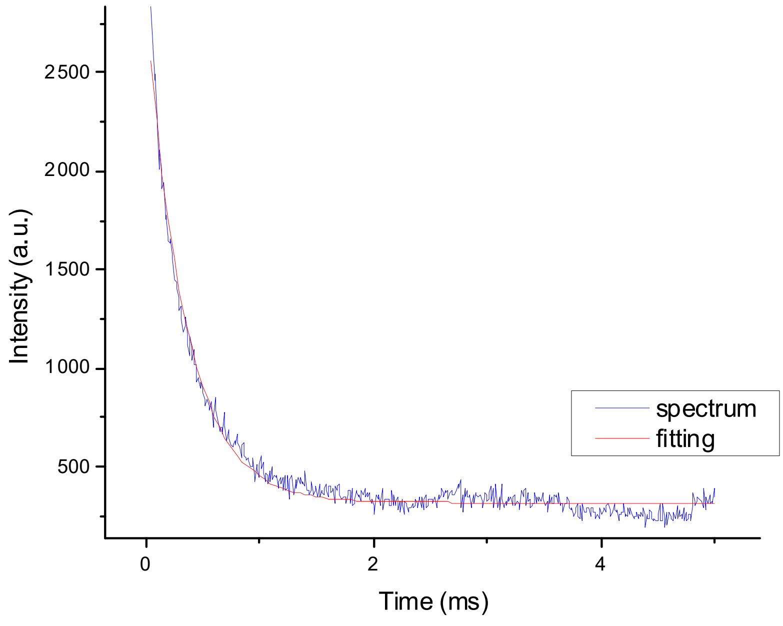

Figure S15. Decay curve and fitting data for complex 1 in the solid state at 77 K (λex = 400 nm; λem = 550 nm)

	y0	y0	A1	A1	t1	t1	Statistics	Statistics
	Value	Standard E	Value	Standard E	Value	Standard E	Reduced C	Adj. R-Squ
S1	248,39161	30,47436	122133,83	144,32625	1,04489	0,00203	54081,291	0,99991

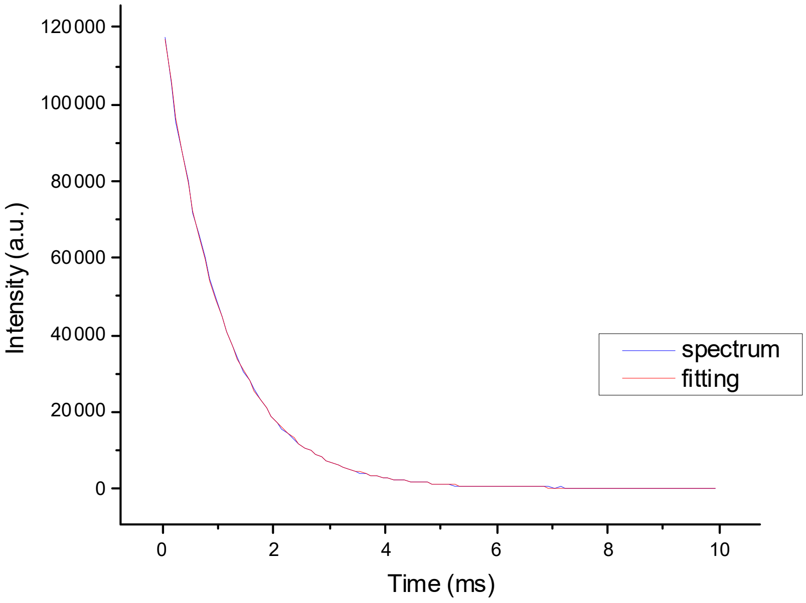

Figure S16. Decay curve and fitting data for complex 1 in 10^{-3} M acetone solution at 77K (λex = 270 nm; λem = 440 nm)

	y0	y0	A1	A1	t1	t1	Statistics	Statistics
	Value	Standard Error	Value	Standard Error	Value	Standard Error	Reduced Chi-Sq	Adj. R-Square
S1	3,6452	0,23908	2819,55161	19,59283	0,02299	1,25574E-4	16,55751	0,99792

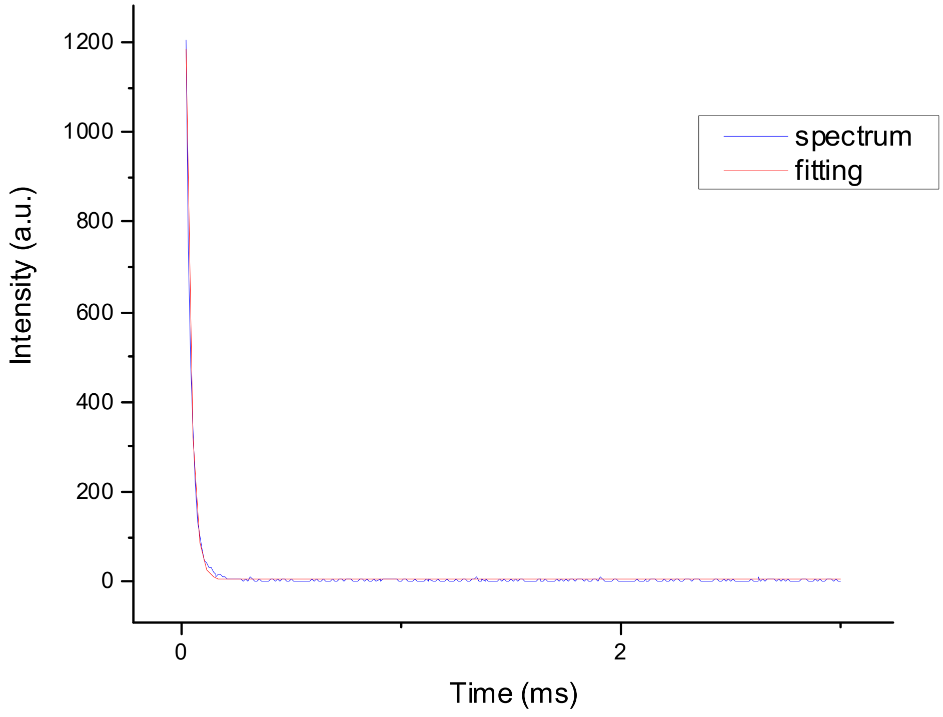

Figure S17. Decay curve and fitting data for complex 2 in film (λex = 395 nm; λem = 542 nm)

	y0	y0	A1	A1	t1	t1	Statistics	Statistics
	Value	Standard Er	Value	Standard Er	Value	Standard Er	Reduced C	Adj. R-Squa
S1	6,98627	3,88337	16535,046	179,61329	0,02575	3,25739E-4	743,93145	0,99934

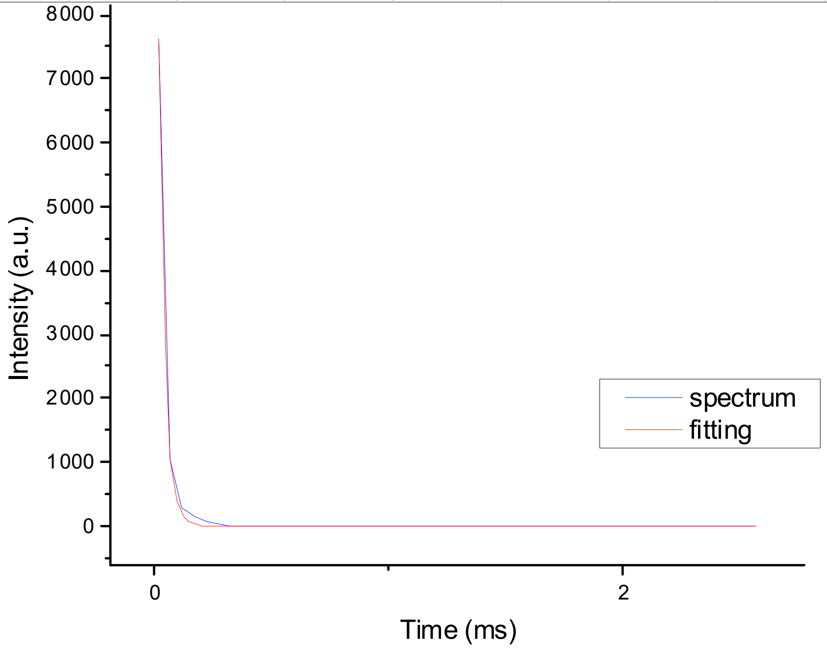

Figure S18. Decay curve and fitting data for complex 2 in the solid state at room temperature (λex = 450 nm; λem = 542 nm)

	y0	y0	A1	A1	t1	t1	Statistics	Statistics
	Value	Standard Err	Value	Standard Err	Value	Standard Err	Reduced Chi	Adj. R-Squar
S1	157,52097	23,77257	18646,4168	115,53077	2,93225	0,02998	52205,3865	0,99633

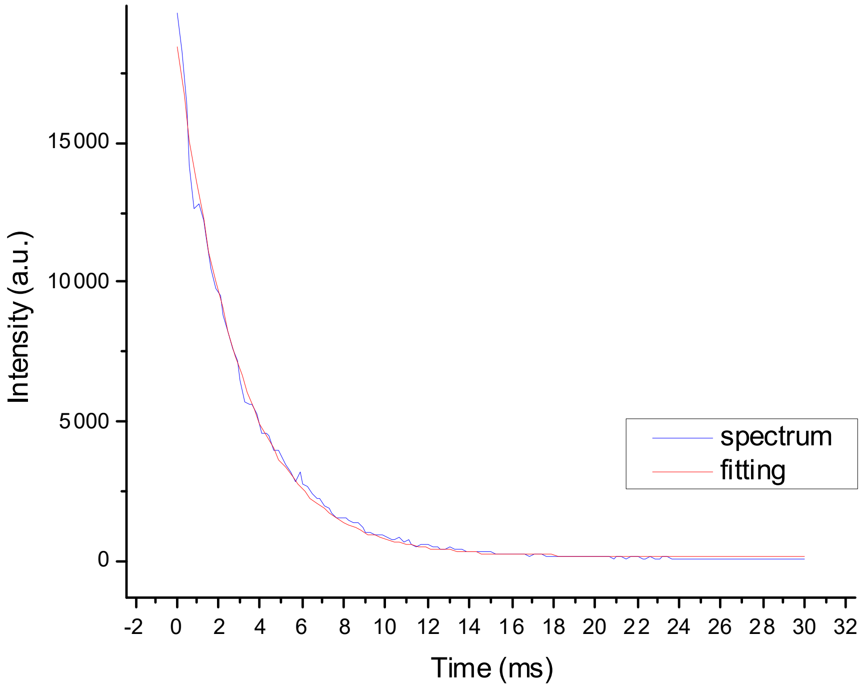

Figure S19. Decay curve and fitting data for complex 2 in the solid state at 77 K (λex = 405 nm; λem = 545 nm)

	y0	y0	A1	A1	t1	t1	Statistics	Statistics
	Value	Standard E	Value	Standard E	Value	Standard E	Reduced C	Adj. R-Squ
S1	571,75839	9,88354	12387,905	38,42274	3,88555	0,02103	14333,889	0,99806

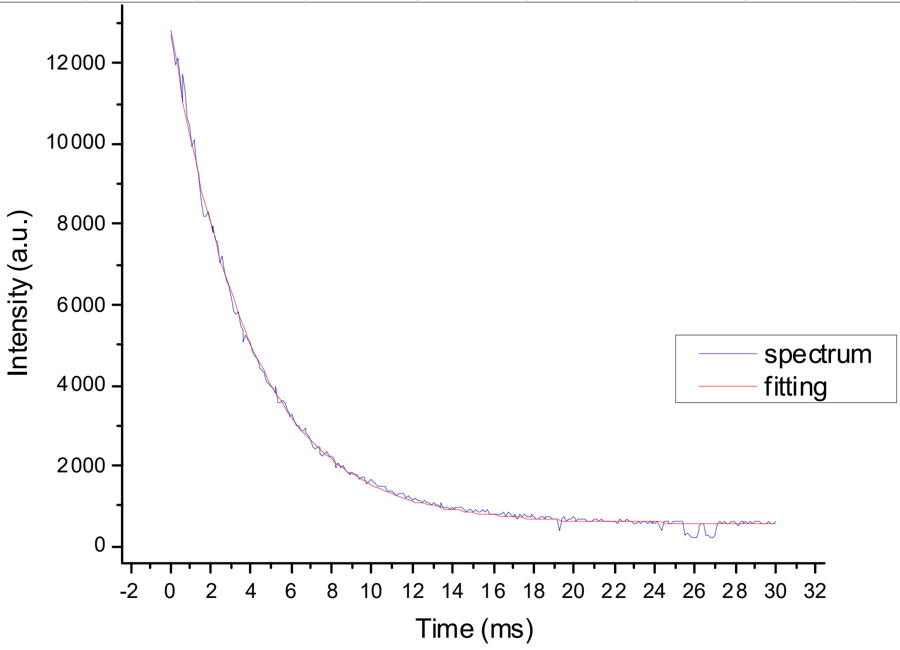

Figure S20. Decay curve and fitting data for complex 2 in 10^{-3} M acetone solution at 77K (λex = 315 nm; λem = 530 nm)

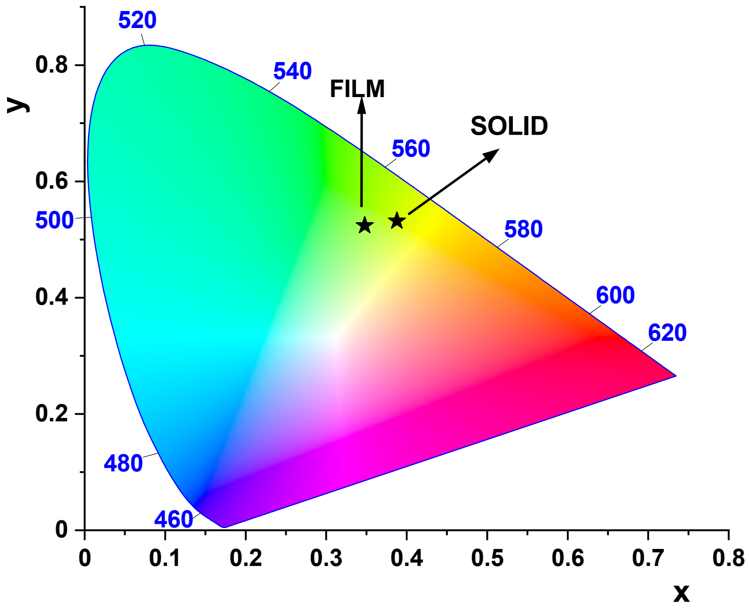

Figure S21. CIE 1931 (angle 2°) diagram of color for complex 2

Table S1. CIE 1931 (angle 2°) coordinates* for 2

**Compound 2**	X	Y
Powder, room temperature	0.388	0.532
PPMA film	0.348	0.524

From the emission spectra after removing excitation light.

Table S2. Energy values for 1 and 2[1]

**Compound 1**	
**Zero-point correction=**	0.679603 (Hartree/Particle)
**Thermal correction to Energy=**	0.724462
**Thermal correction to Enthalpy=**	0.725406
**Thermal correction to Gibbs Free Energy=**	0.599552
**Sum of electronic and zero-point Energies=**	-3082.089944
**Sum of electronic and thermal Energies=**	-3082.045084
**Sum of electronic and thermal Enthalpies=**	-3082.044140
**Sum of electronic and thermal Free Energies=**	-3082.169995

**Compound 2**	
**Zero-point correction=**	0.671737 (Hartree/Particle)
**Thermal correction to Energy=**	0.716322
**Thermal correction to Enthalpy=**	0.717267
**Thermal correction to Gibbs Free Energy=**	0.591039
**Sum of electronic and zero-point Energies=**	-3057.403376
**Sum of electronic and thermal Energies**	-3057.358791
**Sum of electronic and thermal Enthalpies=**	-3057.357846
**Sum of electronic and thermal Free Energies=**	-3057.484074
**Zero-point correction=**	0.671737 (Hartree/Particle)

References

[1] *Results shown in Tables S2 and S3 have been obtained using the program: Gaussian 09, Revision E.01,* M. J. Frisch, G. W. Trucks, H. B. Schlegel, G. E. Scuseria, M. A. Robb, J. R. Cheeseman, G. Scalmani, V. Barone, B. Mennucci, G. A. Petersson, H. Nakatsuji, M. Caricato, X. Li, H. P. Hratchian, A. F. Izmaylov, J. Bloino, G. Zheng, J. L. Sonnenberg, M. Hada, M. Ehara, K. Toyota, R. Fukuda, J. Hasegawa, M. Ishida, T. Nakajima, Y. Honda, O. Kitao, H. Nakai, T. Vreven, J. A. Montgomery, Jr., J. E. Peralta, F. Ogliaro, M. Bearpark, J. J. Heyd, E. Brothers, K. N. Kudin, V. N. Staroverov, T. Keith, R. Kobayashi, J. Normand, K. Raghavachari, A. Rendell, J. C. Burant, S. S. Iyengar, J. Tomasi, M. Cossi, N. Rega, J. M. Millam, M. Klene, J. E. Knox, J. B. Cross, V. Bakken, C. Adamo, J. Jaramillo, R. Gomperts, R. E. Stratmann, O. Yazyev, A. J. Austin, R. Cammi, C. Pomelli, J. W. Ochterski, R. L. Martin, K. Morokuma, V. G. Zakrzewski, G. A. Voth, P. Salvador, J. J. Dannenberg, S. Dapprich, A. D. Daniels, O. Farkas, J. B. Foresman, J. V. Ortiz, J. Cioslowski, and D. J. Fox, Gaussian, Inc., Wallingford CT, **2013**.
